# No Added Neuroprotective Effect of Remote Ischemic Postconditioning and Therapeutic Hypothermia After Mild Hypoxia-Ischemia in a Piglet Model

**DOI:** 10.3389/fped.2020.00299

**Published:** 2020-06-26

**Authors:** Ted C. K. Andelius, Mette V. Pedersen, Hannah B. Andersen, Mads Andersen, Vibeke E. Hjortdal, Michael Pedersen, Steffen Ringgaard, Lærke H. Hansen, Tine B. Henriksen, Kasper J. Kyng

**Affiliations:** ^1^Department of Pediatrics, Aarhus University Hospital, Aarhus, Denmark; ^2^Department of Cardiothoracic and Vascular Surgery, Aarhus University Hospital, Aarhus, Denmark; ^3^Comparative Medicine Lab, Aarhus University Hospital, Aarhus, Denmark; ^4^The MR Research Centre, Aarhus University Hospital, Aarhus, Denmark

**Keywords:** neonatal encephalopathy, neuroprotection, remote ishemic postconditioning, piglet model, magnetic resonance imaging, magnetic resonance spectroscopy

## Abstract

**Introduction:** Hypoxic ischemic encephalopathy (HIE) is a major cause of death and disability in children worldwide. Apart from supportive care, the only established treatment for HIE is therapeutic hypothermia (TH). As TH is only partly neuroprotective, there is a need for additional therapies. Intermittent periods of limb ischemia, called remote ischemic postconditioning (RIPC), have been shown to be neuroprotective after HIE in rats and piglets. However, it is unknown whether RIPC adds to the effect of TH. We tested the neuroprotective effect of RIPC with TH compared to TH alone using magnetic resonance imaging and spectroscopy (MRI/MRS) in a piglet HIE model.

**Methods:** Thirty-two male and female piglets were subjected to 45-min global hypoxia-ischemia (HI). Twenty-six animals were randomized to TH or RIPC plus TH; six animals received supportive care only. TH was induced through whole-body cooling. RIPC was induced 1 h after HI by four cycles of 5 min of ischemia and 5 min of reperfusion in both hind limbs. Primary outcome was Lac/NAA ratio at 24 h measured by MRS. Secondary outcomes were NAA/Cr, diffusion-weighted imaging (DWI), arterial spin labeling, aEGG score, and blood oxygen dependent (BOLD) signal measured by MRI/MRS at 6, 12, and 24 h after the hypoxic-ischemic insult.

**Results:** All groups were subjected to a comparable but mild insult. No difference was found between the two intervention groups in Lac/NAA ratio, NAA/Cr ratio, DWI, arterial spin labeling, or BOLD signal. NAA/Cr ratio at 24 h was higher in the two intervention groups compared to supportive care only. There was no difference in aEEG score between the three groups.

**Conclusion:** Treatment with RIPC resulted in no additional neuroprotection when combined with TH. However, insult severity was mild and only evaluated at 24 h after HI with a short MRS echo time. In future studies more subtle neurological effects may be detected with increased MRS echo time and post mortem investigations, such as brain histology. Thus, the possible neuroprotective effect of RIPC needs further evaluation.

## Introduction

Neonatal hypoxic ischemic encephalopathy (HIE) is a major cause of death and impairment in children (1). Treatment with therapeutic hypothermia (TH) has improved outcome in neonates with moderate to severe HIE, but morbidity and mortality remain high (2). Treatment with TH is limited by a narrow therapeutic time window and further limited to tertiary centers due to technical requirements and the need for specially trained staff. Accordingly, there is a need for neuroprotective strategies that can be combined with TH to improve outcome.

In 1986, Murry et al. showed in dogs that infarction size after coronary occlusion was reduced if the acute myocardial injury was preceded by short ischemic periods—preconditioning (3). Through short periods ischemia applied to a hind limp after the insult, the similar tissue protective effect was later demonstrated in a stroke model in adult rats—remote ischemic postconditioning (RIPC) (4). The tissue protective mechanism of RIPC remains to be fully elucidated together with the optimal timing, number, and duration of remote ischemic cycles. In a review of RIPC for cardio and neuro protection, clinical studies used 5-min cycles of RIPC (5, 6). RIPC has been proposed as a novel neuroprotective intervention for HIE and proven to be neuroprotective in both smaller and larger animal models (7). RIPC reduced infarct volume in rat pups compared to untreated controls (8). Another study in rat pups found improved long-term motor sensory deficits in animals treated with RIPC 24 h after the insult (9). When applied in a larger animal model of HIE, Ezzati et al. found reduced white matter Lac/NAA ratio, higher levels of whole brain ATP, and reduced histological white matter damage with 10 min cycles of RIPC (10). RIPC has also been shown to reduce nitrosative stress in piglets with HIE (11). We have recently shown reduced Lac/NAA ratio in the basal ganglia in piglets treated with 5-min cycles of RIPC compared to piglets who received supportive care only (12). RIPC is a low-tech, readily available intervention and therefore holds potential as a novel neuroprotectant for neonates with HIE. However, it is unknown whether RIPC in combination with TH will improve neuroprotection beyond that of TH alone (13).

We therefore investigated whether there is an added neuroprotective effect of combining RIPC with TH compared to TH alone using magnetic resonance imaging and spectroscopy (MRI/MRS).

## Materials and Methods

The study was approved by the Danish Animal Experiments Inspectorate (Permission nr. 2016-15-0201-01052). This study is reported in accordance with the ARRIVE guidelines (ARRIVE checklist in Supplementary Material 1) (14). Details on this piglet model of HIE have previously been given (12, 15).

### Anesthesia

Newborn Danish Landrace piglets (<12 h old) were used in this study. Animals were transported directly from the farm to the experimental facilities. Piglets were anesthetized using inhalation of 2–4% sevoflurane. Peripheral intravenous access was acquired through an ear vein. A bolus of propofol 10 mg/kg, fentanyl 30 μg/kg, and rocuronium 1 mg/kg was given and the piglet was intubated and ventilated. Anesthesia was maintained through an infusion of propofol 4–10 mg/kg/h and fentanyl 5–12 μg/kg/h. Anesthetics were reduced to the lowest relevant dose to minimize any possible effect on the aEEG and neurological outcomes. The ventilator was adjusted to an end-tidal CO_2_ of 4.5–5.5 kPa. Under sterile conditions, umbilical venous and arterial catheters were placed. SatO_2_%, heart rate, mean arterial blood pressure (MABP), core temperature, and electrocardiogram were continuously recorded and downloaded to a computer (Datex Ohmeda S/5 Collect, Finland). Core temperature was measured through a rectal thermometer placed ~5 cm into the rectum. A single-channel amplitude-integrated electroencephalogram (aEEG) was recorded continuously (Natus Medical Incorporated, CA, USA). Two electrodes were placed on the left and the right side, one behind each eye (approximately equivalent to the parietal 3 and 4 electrodes used in a human neonate). A reference electrode was placed at the base of the snout and a ground electrode on the most caudal part of the head. Piglets received i.v. gentamicin 5 mg/kg once every 24 h and ampicillin 30 mg/kg every 12 h. Blood glucose and electrolytes were monitored and kept within the normal range through infusion of 5–10 ml/kg/h of NeoKNaG (Na^+^: 15 mmol/L, K^+^: 10 mmol/L, Cl^−^: 25 mmol/L, glucose: 505 mmol/L). We aimed to keep MABP >40 mmHg. If hypotension occurred, anesthetics were reduced to the minimum relevant dose and the following treatment given: first, a bolus of saline (10 ml/kg) was administered, followed by infusion of noradrenaline (0.25–1.5 μg/kg/min) and/or dopamine (5–15 μg/kg/min) and/or adrenalin (0.1–1.5 μg/kg/min) and/or dobutamine (2–20 μg/kg/min) (16). For refractory hypotension a bolus of hydrocortisone 2.5 mg/kg was administered.

### Hypoxic-Ischemic Insult

During a 45-min period, piglets were ventilated at an FiO_2_ of 2–10% to mimic the generalized hypoxia neonates may experience during birth. To ensure maximal survival combined with a clinically relevant insult, FiO_2_ was titrated to a target aEEG (<7μV) combined with a target MABP (<70% of baseline MABP) for at least 5 min. FiO_2_ was briefly increased if HR <80 min^−1^ or aEEG <3 μV. After 45 min of hypoxia the piglets were resuscitated at an FiO_2_ of 21%. If needed, FiO_2_ was increased to keep SatO_2_ > 90%.

### Therapeutic Hypothermia

TH was achieved through whole-body cooling with a target temperature of 33.5–34.0°C. TH was induced by active cooling with 5°C water bags directly placed on the piglet until target temperature was reached and then maintained through passive cooling with ambient air. TH was commenced 90 min after HI and continued for 24 h.

### Remote Ischemic Postconditioning

Sixty minutes after the HI insult, RIPC was induced by four conditioning cycles of 5 min of ischemia and 5 min of reperfusion on both hind limbs. Total occlusion of blood flow was induced by two plastic strips around the proximal part of the hind limbs, and absence/presence of blood flow was verified by ultrasound with Doppler. 5-min periods of ischemia/reperfusion have been found cardio- and neuroprotective in adults (5, 6). In accordance with this, we have previously found reduced brain Lac/NAA ratios in piglets treated with 5 min of RIPC compared to untreated controls (12). Thus, to ensure comparability, 5 min of ischemia/reperfusion were chosen for this study.

### Magnetic Resonance Imaging and Spectroscopy (MRI/MRS)

MRI/MRS was performed with a 3 T MR scanner using a knee transmit/receive coil (Skyra, Siemens, Erlangen, Germany). Axial and coronal T2-weighted images were acquired [fast spin echo, repetition and echo time (TR/TE) 6,430/74 ms, slice thickness 2 mm, matrix 320 × 240, field of view (FOV) 160 × 160 mm^2^]. Diffusion-weighted images (DWI) were acquired (single-shot EPI, TR/TE 3,300/108 msec, slice thickness 3 mm, matrix 196 × 190, FOV 213 × 206 mm^2^, b-value 800 s/mm^2^) and apparent diffusion coefficient (ADC) values were calculated in a region of interest (ROI) in the right thalamus. Perfusion-weighted images were acquired using an arterial spin labeling (ASL) sequence (PICORE Q2T, single-shot EPI, TR/TE 3,200/25.6 ms, slice thickness 5 mm, 84 × 84 matrix, FOV 144 × 144 mm^2^), and whole brain perfusion was calculated in three slices and averaged. Due to low signal-to-noise ratio, ASL data may be negative; all negative values were manually removed before final analysis. A multiecho gradient echo (MGRE) sequence was used for obtaining T2^*^ maps (11 echoes TR/TE 431/3.67–49 ms, slice thickness 4 mm, 192 × 126 matrix, FOV 180 × 118 mm^2^). ROIs were drawn on the thalamus in two slices and values were averaged. The researcher performing the MRI data analysis was blinded to treatment allocation in the two intervention groups, but not to the group receiving supportive care only. Images were analyzed with Horos software (Annapolis, MD, USA) version 3.3.5. A representative image is provided in Supplementary Material 2. Single voxel proton MRS (PRESS, TR/TE 2,000/135 ms, voxel size 8 × 8 × 8 mm^3^, 1,024 sample points, spectral width (SW) 1,200 Hz, 128 averages) was acquired in the right side of thalamus, subcortical right-side white matter at the centrum semiovale level, and frontal and occipital cortex. N-acetylaspartate (NAA, 2.02 ppm), lactate (Lac, 1.33 ppm), choline (Cho, 3.2 ppm), and creatine (Cr, 3.02 ppm) were identified. Spectroscopy data were analyzed with LCModel (Stephen Provencher, Oakville, ON, Canada) version 6.3-1L, and Lac/NAA and NAA/Cr ratios were calculated. Thalamic MRS was acquired in two planes and averaged. An image illustrating voxel location and graph can be found in Supplementary Material 2.

### Electroencephalographic Analysis

Amplitude integrated EEG (aEEG) recordings were obtained continuously throughout the study. During transport and acquisition of MRI/MRS, electrodes were removed and recordings were paused. aEEG was recorded for all animals during the HI insult. Due to equipment limitations, aEEG was recorded in only half of the animals during the observation period after the HI insult. aEEG recordings were analyzed and scored from 0 to 4 depending on severity as previously described (17, 18). aEEG recordings (0; flat trace, 1; continuous low voltage, 2; burst suppression, 3; discontinuous normal voltage, and 4; normal voltage) were analyzed for each hour after the HI insult and then averaged at 1, 6, 12, 18, and 24 h. Animals were selected randomly for continuous aEEG recording, and the researchers calculating the aEEG score were blinded to the intervention.

### Experimental Protocol

A total of 26 piglets were subjected to 45 min of global hypoxia-ischemia (HI) and randomized to RIPC + TH (RIPC + TH group) or TH alone (TH group). The experimental protocol was carried out in two piglets from the same litter on each experimental day. After the HI insult one piglet was randomized to one of the intervention groups, while the other was automatically allocated to the other. Six piglets served as controls and were subjected to the HI insult, received supportive care only, and were kept normothermic (NT group). Animals were observed for 24 h and MRI/MRS was performed at 6, 12, and 24 h of observation. After 24 h, piglets were euthanized by a lethal pentobarbital (80 mg/kg) injection.

### Statistics

Based on the data from Zhou et al., a reduction in infarction size from 31 to 22% in RIPC treated animals, and assuming an alpha level of 5 and 90% power, we estimated that 11 animals were sufficient to detect an effect in this study (8). With an expected mortality of 10%, 13 animals were enrolled in each treatment group. Statistical analysis was performed using GraphPad Prism® v8 software. Use of inotropes was registered hourly as infusion rate and then averaged for the 24-h observation period. Number of animals receiving inotropes was reported, the average infusion rate was calculated in the animals that received inotropes. Non-parametric MRS data were log transformed (y=log(y+(1/6)). MRI/MRS and EEG data were tested by mixed-effect model analysis with assumed sphericity and randomly missing values, corrected for multiple comparisons and post-tested with Tukeys test. Demographic- and insult-severity data, inotrope infusion, blood-gas values, and vital parameters were compared with one-way ANOVA for parametric data and Kruskal-Wallis test for non-parametric data. A two-sided *p*-value < 0.05 was considered statistically significant. Demographic- and insult-severity data, inotrope infusion, blood-gas values, and vital parameters are presented as median with interquartile range (IQR). MRI/MRS data are presented as scatter plots with superimposed median and interquartile range.

## Results

### Insult Severity and Survival

Four animals in the TH group, three animals in the RIPC + TH group, and one animal in the NT group died after the HI insult. All died from refractory hypotension except for the one death in the NT group which was caused by mechanical ventilator failure. Thus, nine animals in the TH group, ten animals in the RIPC+TH group, and five animals in the NT group completed the whole study (Table 1). All three groups received a comparable insult with regard to duration of aEEG depression, hypotension, and metabolic acidosis (Tables 2, 3). The two intervention groups received more dopamine than the NT group (Table 2). One animal in the TH and two animals in the TH + RIPC group received a bolus of hydrocortisone. One animal in the NT group received infusion with adrenaline (0.01 μg/kg/min) and dobutamine (0.42 μg/kg/min). Temperature from before to 1 h after the insult was slightly increased but within normal range (Table 3). TH was successfully induced in both intervention groups and resulted in a decreased heart rate (Table 3).

**Table 1:** Survival and gender distribution in the three groups and number of animals who were scanned at the three time points.

	**Time point**			
**Group and sex [*n*, (f/m)]**	**Baseline**	**6-h scan**	**12-h scan**	**24-h scan**
NT	6 (4/2)	5 (4/1)	5 (4/1)	5 (4/1)
TH	13 (6/7)	12 (5/7)	10 (4/6)	9 (4/5)
RIPC + TH	13 (6/7)	11 (4/7)	11 (4/7)	10 (3/7)

*Seven animals died after the hypoxic ischemic insult at different timepoints. One animal in the NT group was excluded after a secondary hypoxic insult due to ventilator failure. NT, no treatment; TH, therapeutic hypothermia; RIPC, remote ischemic postconditioning*.

**Table 2:** Demographic- and insult-severity data for piglets subjected to a HI insult and subsequently treated with TH, TH + RIPC, or supportive care only.

**Weight (kg)**	
NT	1.8 (1.6–2.4)
TH	1.8 (1.6–1.9)
RIPC + TH	1.7 (1.7–1.9)
**Time <7 uV aEEG (min)**	
NT	26.8 (23.0–29.7)
TH	26.5 (19.4–29.9)
RIPC + TH	28.9 (20.3–37.1)
**Time with MABP <70% of baseline (min)**	
NT	7.8 (4.3–11.3)
TH	7.5 (1.0–14.0)
RIPC + TH	6.8 (2.2–12.8)
**Noradrenaline infusion (μg/kg/min)**	
NT	2/6 [0.39 (0.19–0.60)]
TH	7/13 [0.39 (0.26–0.94)]
RIPC + TH	7/13 [0.40 (0.06–0.75)]
**Dopamine infusion (μg/kg/min)**	
NT	0/6
TH	4/13 [4.17 (3.08–5.00)] #
RIPC + TH	4/13 [4.75 (1.90–7.82)] #

Use of inotropes was registered hourly as infusion rate and then averaged for the 24-h observation period. Number of animals receiving inotropes was reported and for those who received inotropes the average infusion rate calculated. Data are median with interquartile range. One-way ANOVA and Kruskal-Wallis test. # indicates significance vs. NT. ^*^Indicates significance vs. TH or TH + RIPC. HI, hypoxia ischemia; TH, therapeutic hypothermia; NT, no treatment; RIPC, remote ischemic postconditioning; aEEG, amplitude integrated electroencephalography; MABP, Mean arterial blood pressure.

**Table 3:** Arterial blood-gas values and vital parameters at baseline, during hypoxia, and in recovery in piglets subjected to a hypoxic-ischemic insult and subsequently treated with TH, TH + RIPC, or supportive care only.

	**Baseline**	**Hypoxia**			**Recovery**					
		**15 min**	**30 min**	**45 min**	**1 h**	**2 h**	**4 h**	**6 h**	**12 h**	**24 h**
**pH**										
NT	7.54 (7.51–7.56)	7.40 (7.38–7.42)	7.12 (7.10–7.22)	6.97 (6.90–7.06)	7.31 (7.14–7.42)	7.46 (7.35–7.53)	7.43 (7.40–7.50)	7.43 (7.39–7.49)	7.56 (7.49–7.57)	7.45 (7.38–7.49)
TH	7.52 (7.50–7.57)	7.38 (7.33–7.42)	7.22 (7.04–7.28)	7.01 (6.90–7.13)	7.37 (7.25–7.46)	7.44 (7.36–7.48)	7.43 (7.39–7.53)	7.51 (7.40–7.53)	7.51 (7.43–7.57)	7.47 (7.38–7.52)
TH + RIPC	7.55 (7.51–7.58)	7.36 (7.28–7.44)	7.16 (7.09–7.25)	7.06 (6.96–7.11)	7.39 (7.31–7.47)	7.47 (7.40–7.53)	7.49 (7.46–7.52)	7.53 (7.46–7.57)	7.46 (7.45–7.58)	7.49 (7.44–7.54)
**pCO**_**2**_ **(kPa)**										
NT	4.86 (4.28–5.49)	5.38 (4.58–5.69)	5.46 (4.84–6.01)	5.68 (5.11–6.67)	4.73 (3.94–6.42)	4.59 (4.04–5.63)	5.54 (4.89–5.90)	5.78 (5.49–6.52)	4.85 (3.88–4.97)	5.72 (5.18–5.99)
TH	4.91 (4.35–5.14)	4.99 (4.68–5.54)	5.61 (4.00–6.16)	5.72 (4.40–6.59)	4.34 (3.84–5.39)	4.99 (4.46–5.37)	4.91 (4.32–6.19)	5.09 (4.61–5.53)	4.63 (3.98–5.95)	4.96 (4.51–5.42)
TH + RIPC	4.68 (4.35–5.26)	5.67 (4.26–6.28)	5.42 (4.94–6.24)	5.10 (4.80–6.67)	4.12 (3.66–5.43)	4.49 (4.31–5.69)	5.12 (4.55–5.40)	5.26 (4.11–5.73)	4.70 (4.46–5.75)	5.41 (5.18–5.66)
**pO**_**2**_ **(kPa)**										
NT	12.70 (10.88–14.13)	2.06 (1.79–2.26)	2.48 (2.29–4.25)	3.49 (3.14–4.41)	15.35 (13.63–17.48)	15.60 (14.70–19.68)	15.85 (15.35–16.65)	15.70 (11.16–18.78)	16.00 (13.25–17.45)	13.40 (11.85–15.35)
TH	11.70 (10.40–13.30)	1.94 (1.72–2.24)	2.29 (1.99–2.84)	2.68 (2.03–3.90)	12.40 (10.85–14.50)	12.45 (11.43–15.68)	11.80 (10.60–14.00)	11.50 (10.30–13.90)	**11.70 (10.10–13.00) #**	12.20 (10.98–14.53)
TH + RIPC	12.05 (11.35–13.50)	1.90 (1.76–2.60)	2.45 (1.85–3.23)	3.35 (3.08–4.32)	13.80 (11.15–15.85)	**12.35 (9.46–13.45) #**	**10.10 (9.39–12.80) #**	12.30 (11.10–13.50)	**11.40 (10.60–13.60) #**	10.10 (8.79–13.90)
**Lactate (mmol/L)**										
NT	1.70 (0.85–2.15)	9.00 (7.80–10.83)	16.50 (14.73–18.25)	20.50 (19.25–23.50)	11.00 (9.50–13.55)	4.10 (3.40–5.65)	1.45 (1.15–2.50)	1.25 (1.05–1.43)	1.10 (0.85–1.65)	0.90 (0.65–2.20)
TH	1.30 (0.95–1.45)	8.90 (5.70–9.75)	14.30 (12.05–18.00)	19.00 (15.30–23.00)	10.80 (7.45–14.00)	2.90 (1.38–9.00)	0.90 (0.50–1.60)	0.60 (0.60–1.30)	0.90 (0.80–1.40)	0.65 (0.48–0.95)
TH + RIPC	1.40 (1.10–1.68)	9.20 (7.40–9.85)	15.00 (13.70–16.50)	18.00 (17.50–21.00)	9.80 (6.75–11.60)	3.80 (2.70–4.60)	1.10 (0.70–1.90)	0.90 (0.70–1.10)	0.80 (0.70–1.70)	0.90 (0.70–1.18)
**Base Excess (mmol/L)**										
NT	7.75 (4.98–13.65)	−1.80 (−3.05 to −1.53)	−15.00 (−16.25 to −12.33)	−22.75 (−23.85 to −18.05)	−7.05 (−14.00 to −3.05)	0.00 (−3.70 to −6.80)	4.50 (0.48–6.58)	5.35 (0.18–10.05)	6.70 (1.85–9.45)	6.50 (−1.45–8.50)
TH	6.40 (4.65–9.10)	−2.10 (−5.70 to −0.45)	−12.00 (−16.50 to −10.35)	−20.80 (−25.00 to −16.50)	−6.50 (−12.5 to −2.10)	1.00 (−4.73 to −4.25)	3.70 (0.00–5.10)	6.30 (4.10–7.40)	5.20 (3.00–6.40)	3.25 (1.13–6.73)
TH + RIPC	7.80 (6.65–10.23)	−1.80 (−5.30–0.75)	−12.10 (−15.95 to −10.45)	−18.70 (−20.35 to −16.65)	−3.80 (−6.45 to −1.40)	3.35 (−1.55 to −4.50)	5.70 (4.40–7.40)	6.20 (5.10–9.40)	6.00 (3.30–8.70)	8.70 (4.80–10.45)
**Glucose (mmol/L)**										
NT	6.90 (5.65–7.78)	8.15 (7.33–9.10)	11.05 (9.30–13.03)	12.45 (9.98–14.13)	8.75 (5.70–9.83)	6.75 (5.00–7.83)	6.95 (5.80–7.35)	7.10 (6.10–7.55)	4.70 (3.90–5.35)	6.10 (3.85–8.80)
TH	7.10 (5.95–8.80)	6.50 (5.40–7.35)	**8.20 (6.25–9.43) #**	**7.60 (6.20–9.15) #**	7.50 (6.15–8.85)	6.95 (5.30–8.38)	6.70 (5.30–10.00)	6.50 (5.30–11.00)	7.60 (6.90–9.10)	7.95 (3.83–10.08)
TH + RIPC	7.30 (6.85–9.85)	6.90 (6.30–9.90)	8.30 (7.30–11.15)	8.90 (7.45–11.60)	6.80 (6.20–8.50)	6.40 (4.60–6.60)	6.10 (5.00–8.90)	5.70 (5.10–9.10)	**9.80 (6.10–12.20) #**	8.40 (6.45–12.65)
**Heart rate (bpm)**										
NT	112 (115–167)	182 (159–210)	157 (131–218)	166 (135–204)	172 (141–218)	184 (152–250)	214 (154–250)	213 (173–250)	189 (155–224)	190 (156–219)
TH	136 (127–149)	207 (197–222)	176 (163–197)	182 (156–218)	182 (169–213)	143 (122–179)	**126 (92–180) #**	**127 (110–175) #**	**120 (117–148) #**	157 (127–169)
TH + RIPC	134 (125–142)	200 (187–225)	180 (167–200)	165 (147–222)	181 (169–200)	162 (143–174)	**125 (85–143) #**	**116 (91–165) #**	**118 (90–156) #**	**126 (99–158) #**
**Mean arterial blood pressure (mmHg)**										
NT	56 (50–60)	59 (52–59)	39 (34–48)	42 (31–52)	51 (47–57)	51 (43–59)	54 (47–55)	50 (47–56)	53 (46–59)	45 (40–51)
TH	48 (45–57)	50 (47–63)	36 (31–43)	37 (29–50)	47 (44–53)	45 (40–51)	44 (42–51)	45 (41–46)	48 (37–53)	47 (42–50)
TH + RIPC	47 (43–51)	49 (42–56)	39 (31–41)	33 (27–35)	45 (39–49)	45 (43–49)	44 (42–50)	46 (43–49)	48 (44–51)	47 (38–55)
**Rectal temperature (°C)**										
NT	38.6 (37.0–39.2)				39.6 (38.6–40.0)	38.9 (38.5–39.4)	39.3 (38.2–39.5)	39.0 (38.3–39.8)	39.1 (38.2–39.5)	39.0 (38.6–39.6)
TH	38.6 (38.3–39.0)				39.4 (39.0–39.6)	**37.0 (36.2–37.8) #**	**33.9 (33.7–34.1) #**	**33.8 (33.4–34.3) #**	**33.8 (33.7–34.0) #**	**33.8 (33.2–34.0) #**
TH + RIPC	38.5 (38.4–38.9)				39.3 (39.0–39.7)	**37.1 (36.5–37.5) #**	**34.0 (33.7–34.1) #**	**33.9 (33.7–34.3) #**	**34.0 (33.9–34.3) #**	**33.7 (33.6–34.2) #**

Data are median with interquartile range. One-way ANOVA and Kruskal-Wallis test. Values with significant in-group differences are bold. # indicates significance vs. NT. *indicates significance vs. TH or TH + RIPC. TH, therapeutic hypothermia; NT, no treatment; RIPC, remote ischemic postconditioning.

### Magnetic Resonance Spectroscopy

**Figure 1:**
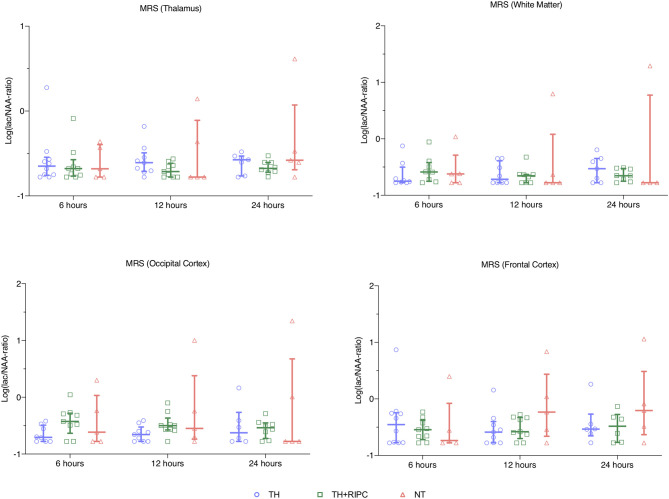
Magnetic resonance spectroscopy in piglets subjected to a HI insult and treated with therapeutic hypothermia (TH), TH and remote ischemic postconditioning (TH + RIPC), or supportive care only (NT). Scans were performed at 6, 12, and 24 h after the insult. Lac, lactate; NAA, N-acetylaspartate. Data are scatter plots with superimposed median and interquartile range. Mixed-effect model analysis, *p < 0.05.

**Figure 2:**
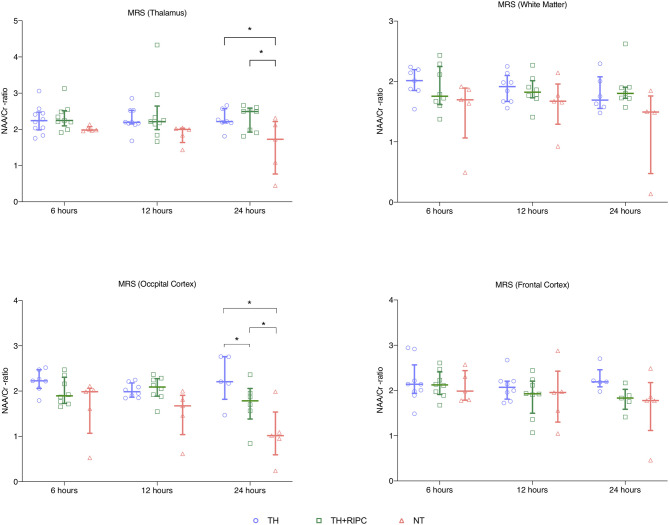
Magnetic resonance spectroscopy in piglets subjected to a HI insult and treated with therapeutic hypothermia (TH), TH and remote ischemic postconditioning (TH + RIPC), or supportive care only (NT). Scans were performed at 6, 12, and 24 h after the insult. NAA, N-acetyl aspartate; Cr, creatinine. Data are scatter plots with superimposed median and interquartile range. Mixed-effect model analysis, *p < 0.05.

MRS showed no difference between the three groups with regards to Lac/NAA ratio at any time point (Figure 1). MRS showed decreased NAA/Cr ratio in the occipital cortex and thalamus 24 h after the insult in the NT group compared to the TH and TH + RIPC group (Figure 2) and the NAA/Cr ratio in the occipital cortex was lower in the TH + RIPC group compared to the TH group (Figure 2). NAA/Cr ratio was stable over time in the two intervention groups, while the NAA/Cr ratio decreased with time in the NT group (Figure 2).

### Magnetic Resonance Imaging

**Figure 3:**
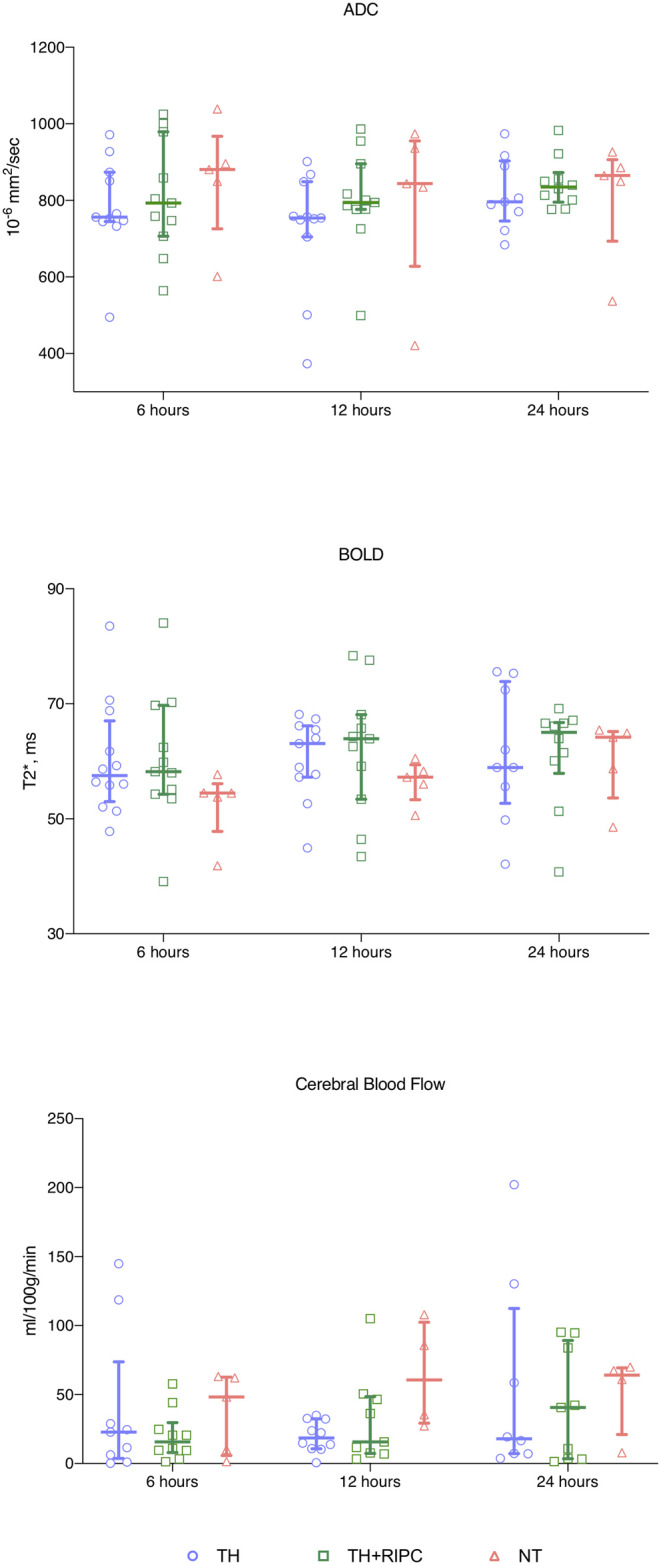
Magnetic resonance imaging in piglets subjected to a HI insult and treated with therapeutic hypothermia (TH), TH and remote ischemic postconditioning (TH + RIPC), or supportive care only (NT). Scans were performed at 6, 12, and 24 h after the insult. ADC, apparent diffusion imaging; BOLD, blood oxygenation level dependent. Data are scatter plots with superimposed median and interquartile range. Mixed-effect model analysis, *p < 0.05.

Cerebral edema measured by DWI showed no difference between the three groups (Figure 3). Although not statistically significant, animals in the NT group had lower cerebral oxygenation 6 and 12 h after the HI insult as assessed from by BOLD measurements (Figure 3). There was no difference between the two intervention groups with regard to cerebral oxygenation. After a quality check of the ASL data one scan was removed from the TH+RIPC group and two from the NT group. CBF measured by ASL was similar in the three groups (Figure 3).

### aEEG Analysis

**Figure 4:**
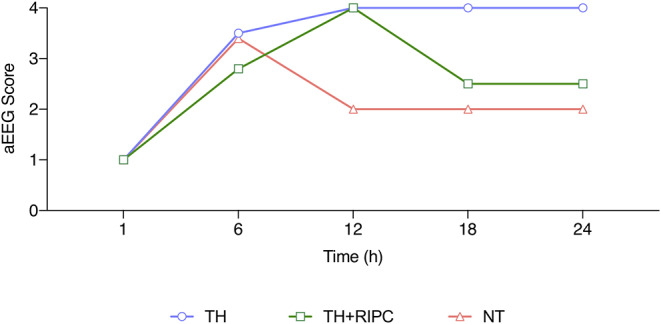
aEEG score for piglets subjected to a HI insult and treated with therapeutic hypothermia (TH), TH and remote ischemic postconditioning (TH + RIPC), or supportive care only (NT). aEEG were scored every hour and averaged for 6, 12, 18, and 24 h. Data are median. Mixed-effect model analysis. ^#^TH vs. NT, *TH vs. TH + RIPC, ^§^NT vs. TH + RIPC.

aEEG was available for three animals in the NT group, six animals in the TH group, and seven animals in the TH+RIPC group. aEEG data were missing during the first 6 h in two animals in the TH + RIPC group. Early EEG recovery (continuous aEEG reached by the 2nd hour) was seen in two of the three animals in the NT group, four of the six animals in the TH group, and two of the five in the TH+RIPC group. Seizures and brief rhythmic discharges were detected in 0 out of three animals in the NT group, two of the six animals in the TH group, and three of the seven animals in the TH + RIPC group. There was no overall difference in aEEG score between the three groups (Figure 4).

## Discussion

This is the first study to assess RIPC in addition to TH in neonatal HIE. Overall, adding RIPC to TH resulted in no additional neuroprotective effect. When comparing duration of aEEG suppression and duration of hypotension from this study with data from a similar study in piglets, the insult severity acquired in this study should be regarded as mild rather than severe (19). Indeed, most of our animals presented with rapid recovery and normalization of aEEG after HI (Figure 4). To ensure survival and substantial neural damage, titration of the insult is essential as too extensive HI results in death and too little HI results in absence of damage (20). Insult titration also ensures that the animal is subjected not only to a period of hypoxia but also to a predetermined period of hypotension. Hypotension combined with hypoxia has been shown to be essential to produce a more severe insult (19). This is in accordance with our own observations in a previous study where severely damaged animals had aEEG suppression of at least 40 min combined with a period of hypotension (12). The importance of cerebral hypoperfusion in the pathology of brain injury is further underlined by other studies in which universal hypoxia was combined with carotid clamping (21). It would therefore be relevant in future studies to investigate the neuroprotective effect of RIPC when combined with TH after a severe insult.

Despite the mild insult, the NT group showed a lower NAA/Cr ratio at 24 h than was seen in the other groups. N-acetylaspartate is an amino acid present in healthy neurons and oligodendrocytes-type 2 astrocyte progenitor cells (22, 23), and a reduction in NAA after a HI insult is due to neural damage and reduction in progenitor cells. Peak-area ratio of NAA/Cr and [NAA] measured by MRS has high prognostic accuracy for neonates with HIE (24, 25). This is in accordance with our results as peak-area ratio of NAA/Cr was reduced as early 24 h after the insult in the NT group. This finding underline one of the possible neuroprotective mechanisms of TH.

Cerebral edema measured by diffusion weighted imaging (DWI) has been proposed as a biomarker of brain injury in neonates with HIE (26). Compared to MRS, DWI has been shown to underestimate the extent of damage when acquired on day 1 (27). McKinstry et al. found that edema measured by diffusion tensor imaging also underestimates the neural damage when performed on day 1 compared to images acquired on day 2–4 (28). In keeping with this, in our study we found no difference between the three groups on ADC maps acquired within the first 24 h after the HI insult.

BOLD measured by MRI is based on the paramagnetic properties of deoxyhemoglobin. Increased concentration of deoxyhemoglobin will result in decreased T2^*^ relaxation time and signal loss (29). In this study, BOLD measurements were performed in the thalamus after the HI insult and compared local differences in oxygenation. Although not statistically significant, animals in the NT group had lower T2^*^ values after 6 and 12 h and reached the same levels as did the TH and TH+RICP groups by 24 h. TH is known to reduce cerebral metabolism (30). The decreased T2^*^ values could indicate an increased turnover of oxyhemoglobin to deoxyhemoglobin due to relatively high metabolism immediately after the HI insult. Increased oxygen consumption is coupled to regional changes in blood flow, and T2^*^ values have been correlated to CBF measured by ASL (31). Surprisingly, change in blood flow was not demonstrated in our study as the NT group presented with CBF values comparable to the two intervention groups despite lower T2^*^ values.

Several of the animals that received TH developed hypotension after the HI insult. Ethical standards for animal studies require pain relief and sedation. A side effect of propofol is reduced cardiac output and hypotension, which may be more severe when propofol is combined with opioids (32). In addition, drug metabolism may be impaired after HI due to liver and kidney failure and then be further reduced by TH because it alters drug metabolism (33, 34). Cardiac function might also be compromised due to TH (35). This was apparent in our study, as piglets in the two TH-treated groups required more inotropes and died from refractory hypotension more often than piglets that received supportive care only. The comparison between the two TH-treated groups should, however, still be valid. The increased mortality due to this complication is unlikely to influence the overall conclusion of this study because the majority of MRI/MRS data were similar between the two TH-treated groups.

## Limitations

Piglets received intravenous infusion with fentanyl and propofol to ensure pain relief and sedation. One of the proposed mechanisms of RIPC is through opioid receptor activation (8). Since both intervention groups received fentanyl, the additional neuroprotective effect of RIPC may have been reduced.

Another limitation is spatial resolution of the MRI data. RIPC was shown to predominantly protect white matter measured by MRS in a previous piglet study (10). Due to limited spatial resolution in the MRI data we were unable to perform DWI, BOLD, and ADC measurements on white matter specifically.

An echo time (TE) of 135 ms was chosen as this is part of the MRS protocol used in our clinical MR Centre. A TE of 135 ms also allows for easy lactate-peak identification due to peak inversion. However, a prolonged TE of 288 ms will increase PRESS detection (36). Lactate peak identification with a TE of 135 ms in a 3T scanner is possibly further complicated by anomalous J-modulation (37). To ensure high spatial resolution, a 8 × 8 × 8 mm voxel was used despite the decrease in signal-to-noise ratio. However, the low signal-to-noise ratio and short TE may have obscured the detection of lactate peaks in some animals. Accordingly, in future studies an TE of 288 ms and a larger voxel size might identify more subtle differences.

We examined MRI/MRS 24 h after HI based on times-series pilot studies that showed significant lactate accumulation detectable early in normothermic piglets. This timing would coincide with the secondary energy failure phase. Clinical data on early MRI/MRS suggest that moderate-severe HIE can be identified at this time (38). However, TH augments and delays neuropathological processes and the possible protective effect of RIPC may not manifest until later as neurological injury after HI is a dynamic process that evolves over time (39). Thus, longer running studies are required to evaluate the full extent of neural damage. We opted to focus on outcome measures available in the clinical setting. However, histological analysis was not performed, but could contribute with valuable information on the more subtle neural damage present at this early stage.

## Conclusion

We found no additional neuroprotective effect when RIPC was added to TH after mild HI. However, despite the mild trauma, the neuroprotective effect of TH was detectable by a reduction in peak-area ratio of NAA/Cr. Our results suggest that future studies may benefit from more severe insults, prolonged TE on MRS, less sedation, histological analysis, and outcomes measured at a later time point after HI.

## Data Availability Statement

The datasets generated for this study are available on request to the corresponding author.

## Ethics Statement

The animal study was reviewed and approved by the Danish Animal Experiments Inspectorate (Permission nr. 2016-15-0201-01052). This study is reported in accordance with the ARRIVE guidelines (ARRIVE checklist in Supplementary Material 1).

## Author Contributions

TA, KK, VH, MP, SR, and TH designed the study. TA, MVP, MA, and HA undertook the experiments. TA, SR, LH, and MP performed data analysis. TA drafted the manuscript. All authors have critically reviewed the drafted manuscript and have approved the final manuscript and agree to be accountable for all aspects of the work.

## Conflict of Interest

The authors declare that the research was conducted in the absence of any commercial or financial relationships that could be construed as a potential conflict of interest.
