# Supplementary material for: No Added Neuroprotective Effect of Remote Ischemic Postconditioning and Therapeutic Hypothermia After Mild Hypoxia-Ischemia in a Piglet Model
Source: Front Pediatr. 2020 Jun 26;8:299. doi: 10.3389/fped.2020.00299 (PMC7333529; doi:10.3389/fped.2020.00299)
Supplement: Supplementary file 1 [file Data_Sheet_1.docx]

**Supplementary material S1**


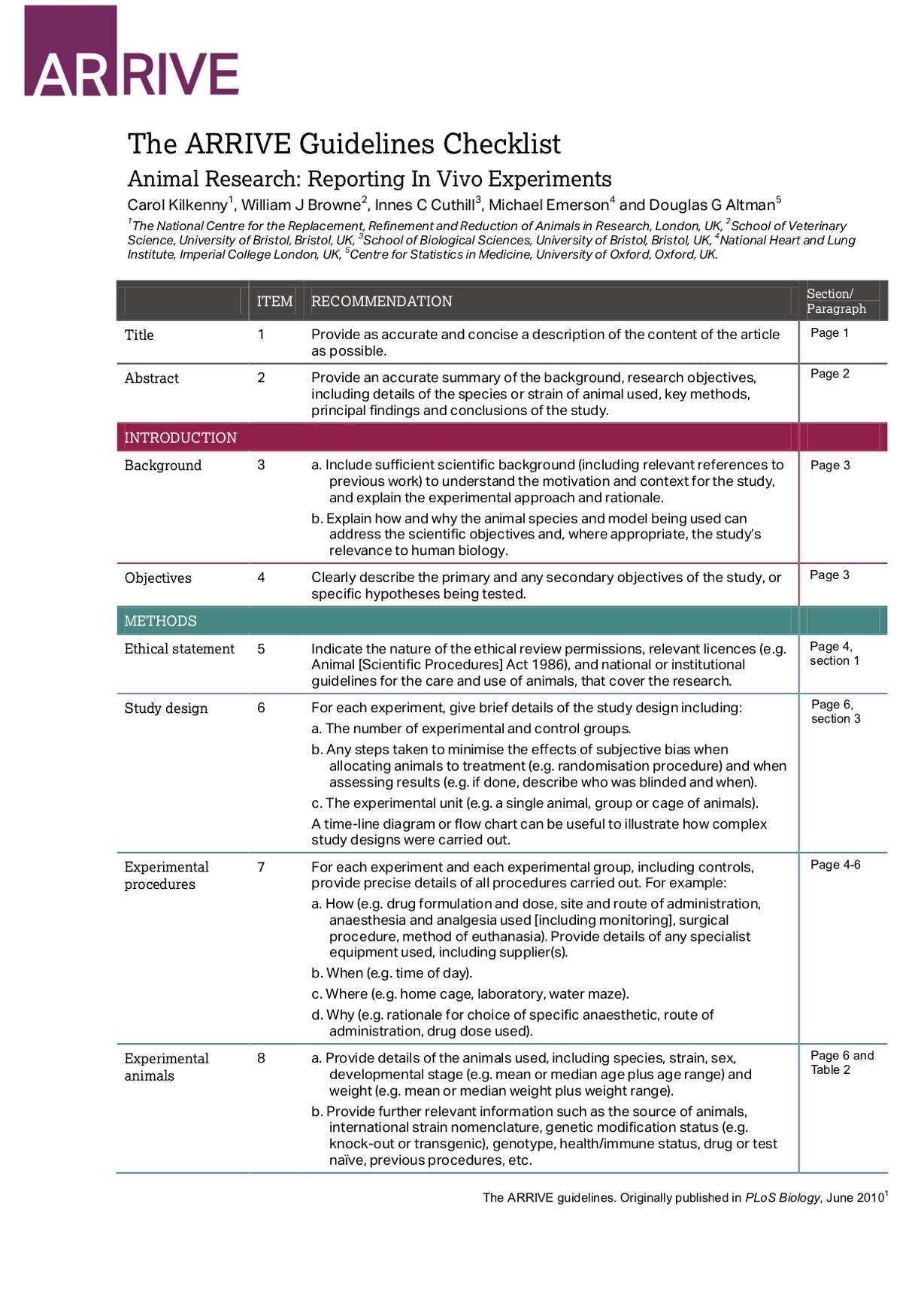


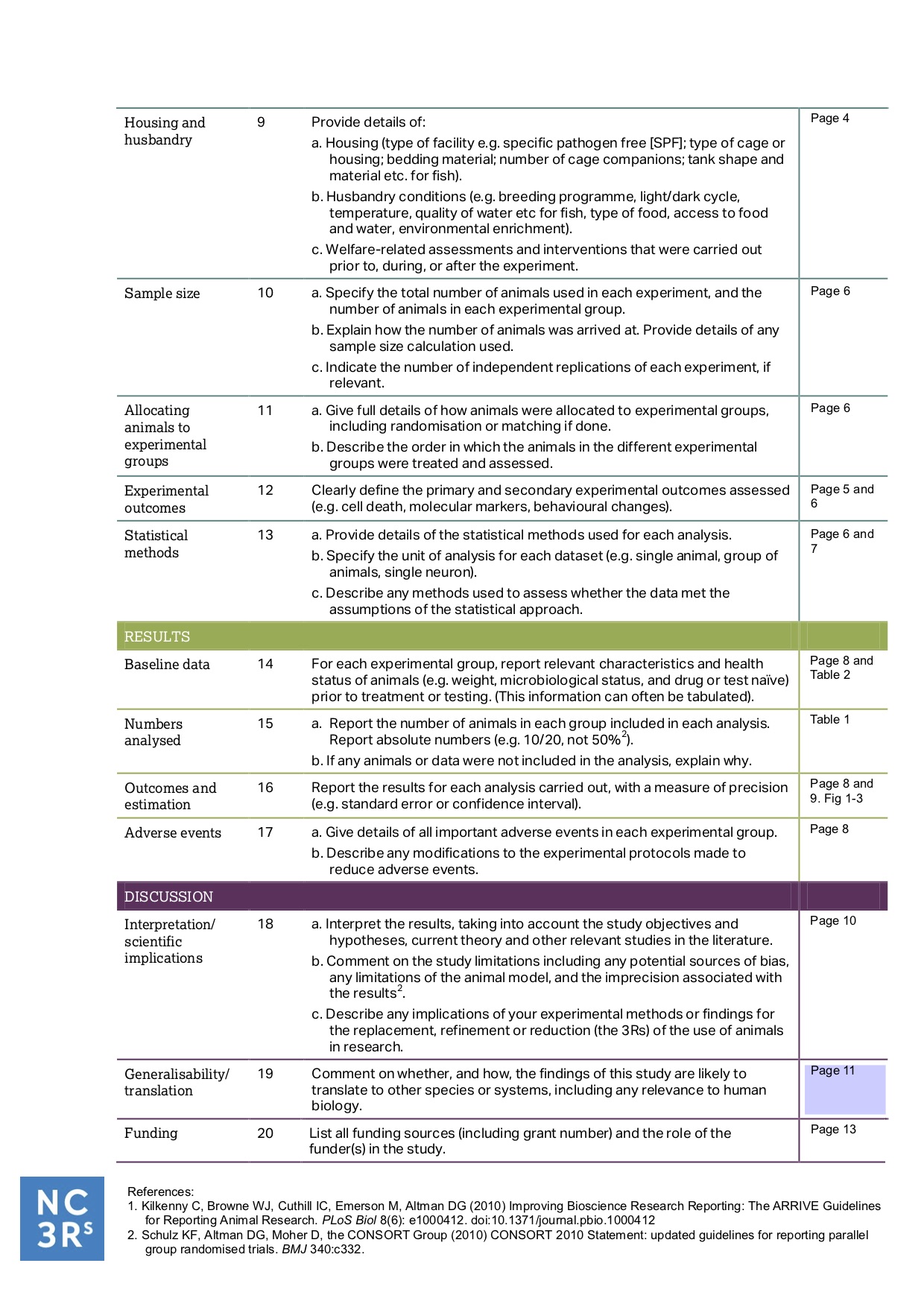


**Supplementary material S2**


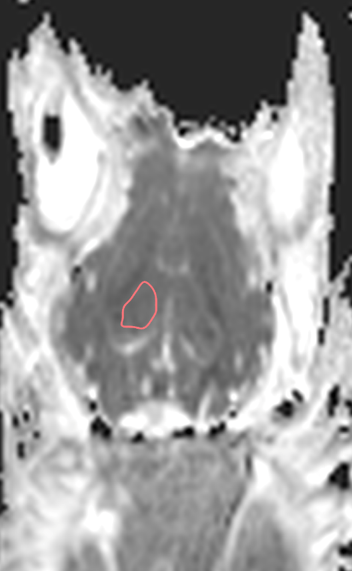

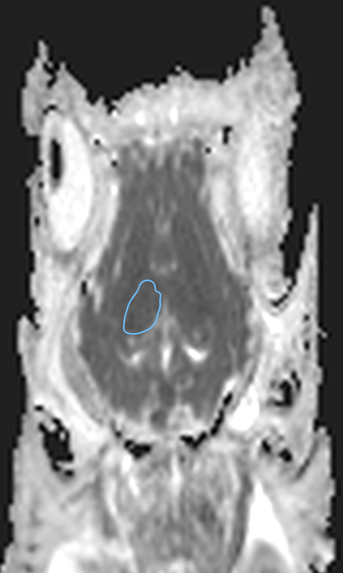

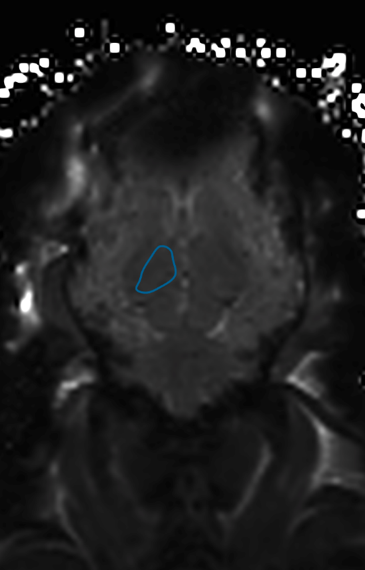

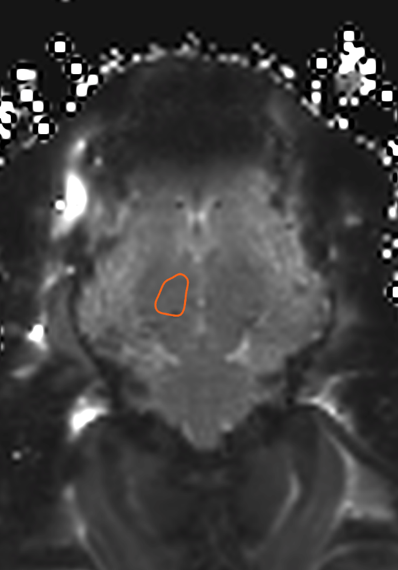

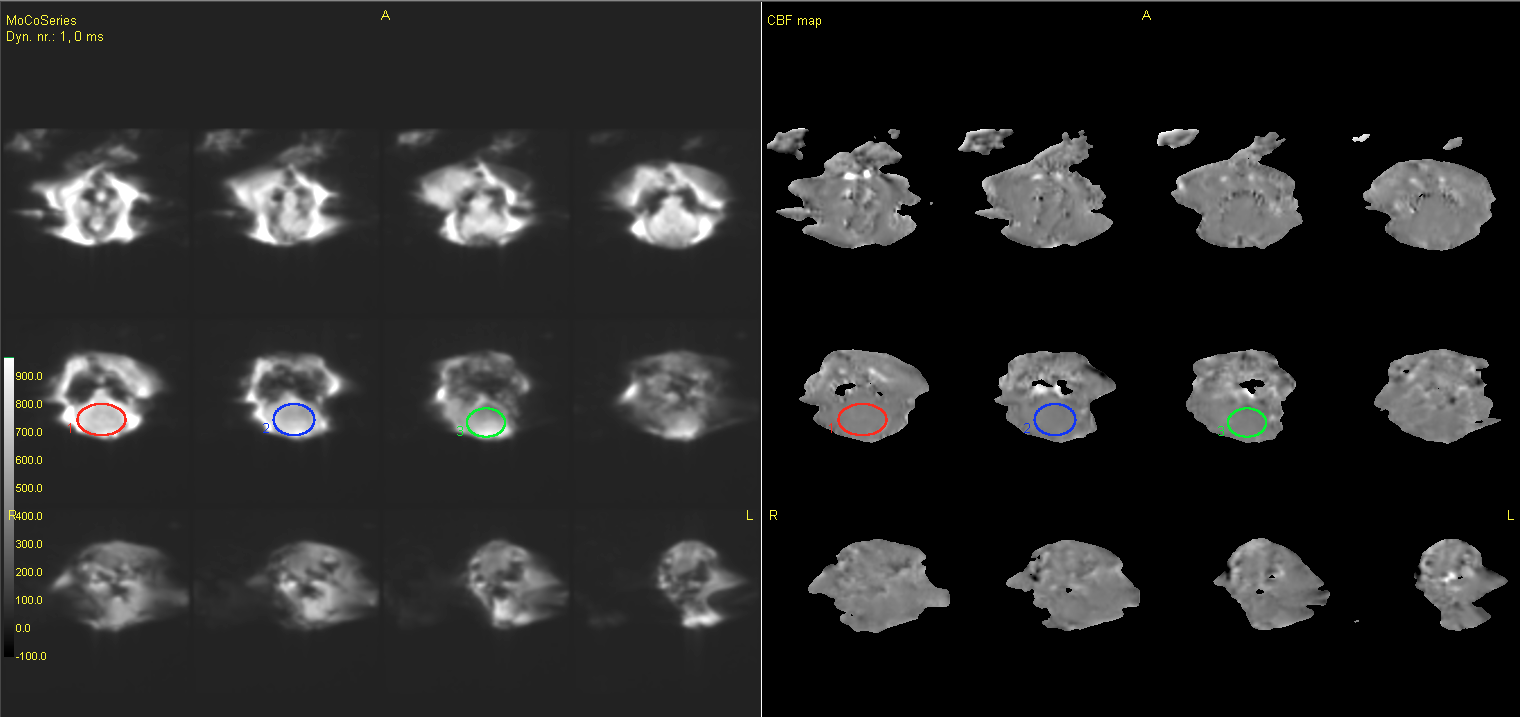


A

B

C

Figure 1. Picture example of ROI location in; A) ADC maps, B) T2*-maps, and C) ASL sequences.

ADC; apparent diffusion imaging. ASL; arterial spin labeling.


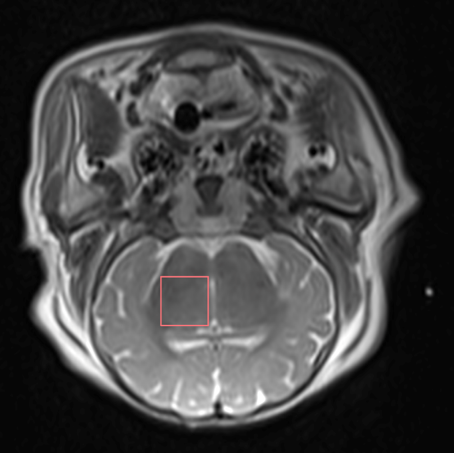

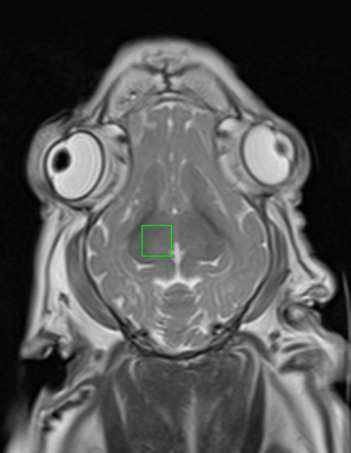

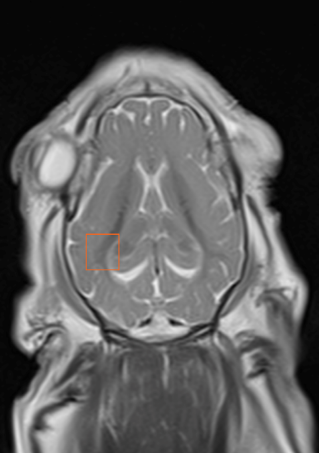

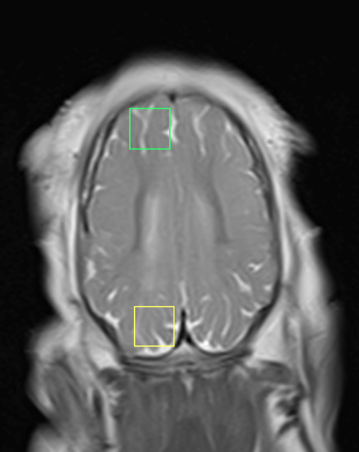


A

B
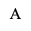


C
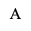


D
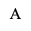


Figure 2. Voxel location in magnetic spectroscopy imaging. A) thalamus in the axial plane, B) thalamus in the coronal plane, C) white matter, D) frontal and occipital cortex. Voxel size in all images is 8x8x8 mm.


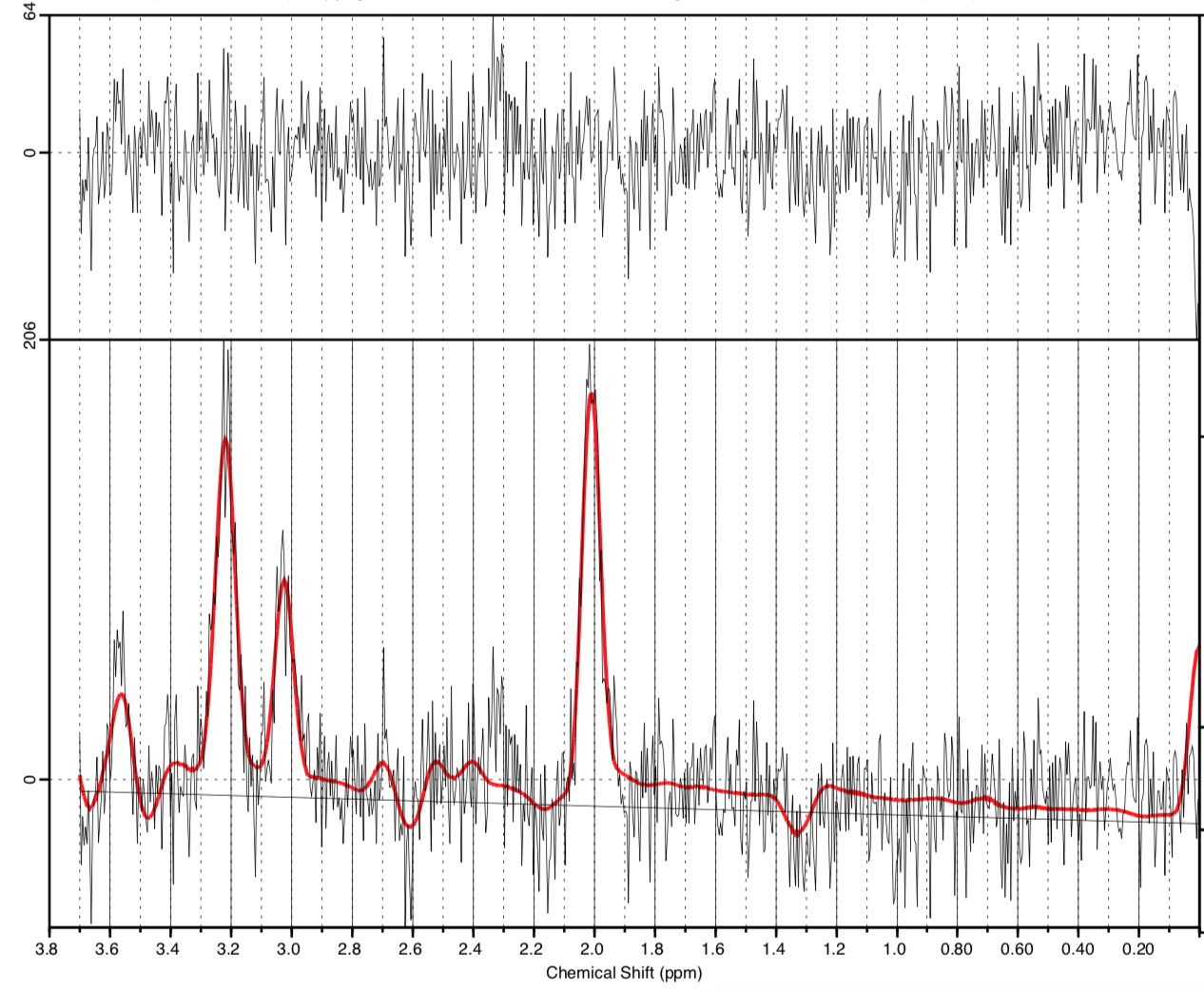


Figure 3. Result of ^1^H-MR data from a piglet subjected to a hypoxic ischemic insult and treated with therapeutic hypothermia. Voxel placement is frontal cortex. Acquired 6 hours after the insult. The lower box shows the spectral curve with a superimposed fitted curve in red. The top graph shows the residual.
